# Case Report: Blinatumomab therapy for the treatment of B-cell acute lymphoblastic leukemia patients with central nervous system infiltration

**DOI:** 10.3389/fimmu.2023.1181620

**Published:** 2023-04-18

**Authors:** Han-Yu Cao, Hui Chen, Song-Bai Liu, Wen-Jie Gong, Chong-Sheng Qian, Tong-Tong Zhang, Chao-Ling Wan, Si-Man Huang, Nan Xu, Hai-Ping Dai, Sheng-Li Xue

**Affiliations:** ^1^ National Clinical Research Center for Hematologic Diseases, Jiangsu Institute of Hematology, The First Affiliated Hospital of Soochow University, Suzhou, China; ^2^ Institute of Blood and Marrow Transplantation, Collaborative Innovation Center of Hematology, Soochow University, Suzhou, China; ^3^ Hematological Department, The First People’s Hospital of Yancheng, Yancheng, China; ^4^ Suzhou Key Laboratory of Medical Biotechnology, Suzhou Vocational Health College, Suzhou, China; ^5^ Research and Development Department, Shanghai Unicar-Therapy Bio-Medicine Technology Co. Ltd, Shanghai, China

**Keywords:** acute lymphoblastic leukemia, chronic myeloid leukemia, blast phase, blinatumomab, central nervous system leukemia, immunotherapy

## Abstract

The treatment of B-cell acute lymphoblastic leukemia (B-ALL) with central nervous system (CNS) involvement poses a significant clinical challenge because most chemotherapeutic agents exhibit weak permeability to the blood-brain barrier (BBB). In addition, current anti-CNS leukemia treatments often bring short or long-term complications. Immunotherapy including chimeric antigen T-cell therapy and bispecific antibody have shown profound treatment responses in relapsed/refractory B-ALL. However, there is a lack of data on the efficacy of bispecific antibody in treating B-ALL with CNS involvement. Here, we report two ALL patients with CNS leukemia who received blinatumomab. Case 1 was diagnosed with chronic myeloid leukemia in lymphoid blast phase. The patient developed CNS leukemia and bone marrow relapse during the treatment with dasatinib. Case 2 was diagnosed with B-ALL and suffered early hematologic relapse and cerebral parenchyma involvement. After treatment with one cycle of blinatumomab, both patients achieved complete remission in the bone marrow and CNS. Furthermore, this is the first report on the efficacy of blinatumomab in treating CNS leukemia with both of the cerebral spinal fluid and the cerebral parenchymal involvement. Our results suggest that blinatumomab might be a potential option for the treatment of CNS leukemia.

## 1 Introduction

The 5-year overall survival (OS) rate of acute lymphoblastic leukemia (ALL) has reached approximately 90% in pediatric and 68% in adult ALL patients with the application of pediatric inspired protocols, targeted and immunotherapies (1). The central nervous system (CNS) is a sanctuary site for ALL cells. According to the literature, approximately 5-10% of ALL patients at diagnosis and 30-40% at relapse are found to have CNS leukemia (2, 3). ALL patients with CNS leukemia had a median OS of only 6 months and a 5-year OS rate of 0, indicating that CNS leukemia remains one of the major causes of treatment failure in ALL (4).

Treatment of CNS leukemia is very challenging due to the impermeability of many systemic therapies to the blood-brain barrier (BBB). CNS-targeted therapy includes intrathecal chemotherapy, cranial radiotherapy, and chemotherapy with high-dose cytarabine or high-dose methotrexate (5). Intrathecal chemotherapy can only penetrate 1-2 mm of tissue and is ineffective when leukemic cells infiltrate deep into the cerebral parenchymal (2). Cranial radiation has the disadvantage of cognitive impairment, pituitary dysfunction, leukoencephalopathy and toxicities to the endocrine system. High-dose chemotherapy is often associated with severe myelosuppression and leukoencephalopathy. What’s more, some patients with CNS leukemia respond poorly to the aforementioned therapies.

Blinatumomab is a CD3/CD19 bispecific antibody that engages autologous T cells to CD19-positive B cells (6), which exerts profound efficacy in B-ALL. The reported complete remission (CR) rate of blinatumomab in relapsed/refractory (R/R) B-ALL was 43.9% (7). Blinatumomab combined with dasatinib yielded 89% of molecular remission at 12-month in newly diagnosed Ph positive B-ALL (8). Another case showed a patient who failed the treatment with chemotherapy and ponatinib. After treatment with blinatumomab, this patient attained major molecular response with a negative T315I mutation (9). At present, there is no report on the application of blinatumomab in the treatment of CNS leukemia. Recently, we used blinatumomab to treat two B-ALL patients with concurrent hematologic relapse and CNS leukemia. To our surprise, both patients achieved complete hematologic and CNS remission.

## 2 Case presentation 1

**Figure 1:**
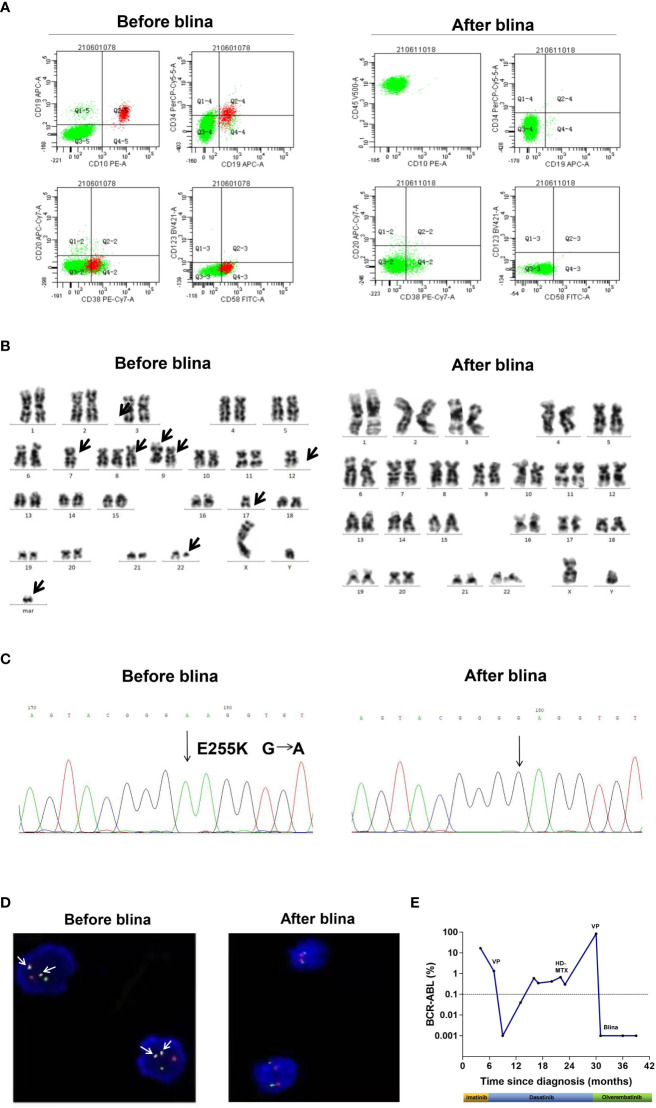
Clinical data of case 1. **(A)** Flow cytometry analysis of cerebral spinal fluid (CSF) before and after blinatumomab (blina). **(B)** G banded chromosome analysis before and after blina. **(C)** Sanger sequencing of ABL1 kinase domain before and after blina. **(D)** Fluorescence *in situ* hybridization (FISH) analysis of *BCR::ABL1* fusion before and after blina. **(E)** Timeline of treatments and responses of case 1. VP, vincristine 1.5 mg/m^2^ intravenously, per week for 4 times and prednisolone 1 mg/kg, once daily, for 4 weeks; HD-MTX, MTX 3.5 g/m^2^, intravenously, for one day.

A 58-year-old male presented with fatigue in June, 2019. Peripheral blood (PB) counts showed white blood cells 67 × 10^9^/L, hemoglobin 127 g/L, platelets 121 × 10^9^/L. Bone marrow (BM) smear showed marked proliferation of the granulocytic lineage. The karyotype was 46, XY, t(9;22)(q34;q11) [20]. P210 *BCR::ABL1* fusion gene (b2a2/b3a2 type) was detected by PCR. The patient was diagnosed with chronic myeloid leukemia in chronic phase (CML-CP). Imatinib at a dose of 400 mg once daily was initiated. The 3-month evaluation result was warning with the *BCR::ABL1* transcripts of 16.61%. However, the patient refused to change to a second-generation tyrosine kinase inhibitor. At 6-month evaluation, BM revealed 39% blasts, which were CD10+/CD19+/CD20+/CD22+ by flow cytometry (FCM). No *ABL1* mutations were detected. Therefore, the disease progressed to lymphoid blast phase. Dasatinib was administered at a dose of 100 mg once daily combined with vincristine (2.5 mg/m^2^ intravenously, per week for 4 times) and prednisolone (1 mg/kg, once daily, for 4 weeks). After completion of the treatment, hematologic CR was achieved. Dasatinib was continued and the *BCR::ABL1* transcripts were 0.18-0.32%. In May 2021, the patient experienced blurred vision. Though the brain MRI was normal, massive blasts (64.2%) were detected in the cerebral spinal fluid (CSF), which were CD10+/CD19+/CD20±/CD22+ by FCM. Fluorescence *in situ* hybridization (FISH) revealed that these blasts were *BCR::ABL1* positive. And *BCR::ABL1* transcripts in the PB increased to 0.67%. Although a complex karyotype and heterozygous *E255K* mutation were observed, the BM morphology remained CR. A diagnosis of CNS leukemia (CNS-3) was established according to NCCN Guidelines for ALL (10). According to the National Institutes of Health Stroke Scale (NIHSS), the patient got 1 point in integrity of visual fields. The final NIHSS score was 1 (11). Dasatinib was continued. Meanwhile, intermittent intrathecal chemotherapies with methotrexate, cytarabine and dexamethasone were applied. High-dose methotrexate (3.5 g/m^2^, intravenously, for one day) were administrated. However, the patient suffered acute renal failure and recovered through hemodialysis. The CNS leukemia was relieved for 5 months but relapsed in Dec, 2021. Blasts from the CSF were CD19 positive as detected by FCM and were positive for *BCR::ABL1* fusion by FISH (**Figures 1A, D**). Meanwhile, a second hematologic relapse was confirmed with 87% blasts in the BM, which were CD10+/CD19+/CD20+/CD22+ by FCM. The *BCR::ABL1* transcripts increased to 83.5%. A complex karyotype, a homozygous E255K and *IKZF1* R511E mutations were observed in the BM (**Figures 1B, C**). Blinatumomab (9 µg d1-7, 28 µg d8-28) was administered combined with olverembatinib (40 mg, every other day). After completion of the treatment, no blasts were detected in the BM as well as CSF by morphology and FISH. Measurable residual disease (MRD) of the BM detected by FCM was <1.0×10^-4^. FCM of CSF showed that no blasts were detected. The karyotype was normal and ABL1 E255K / *IKZF1* mutations were negative. The *BCR::ABL1* transcripts vanished, indicating a deep molecular response. The NIHSS score decreased to 0. Another cycle of blinatumomab plus olverembatinib was administered. The patient did not receive allo-HSCT because he has no suitable donors. Olverembatinib was continued. The patient has remained in deep molecular response for 1 year. The treatment process of case 1 is depicted in **Figure 1E**.

## 3 Case presentation 2

**Figure 2:**
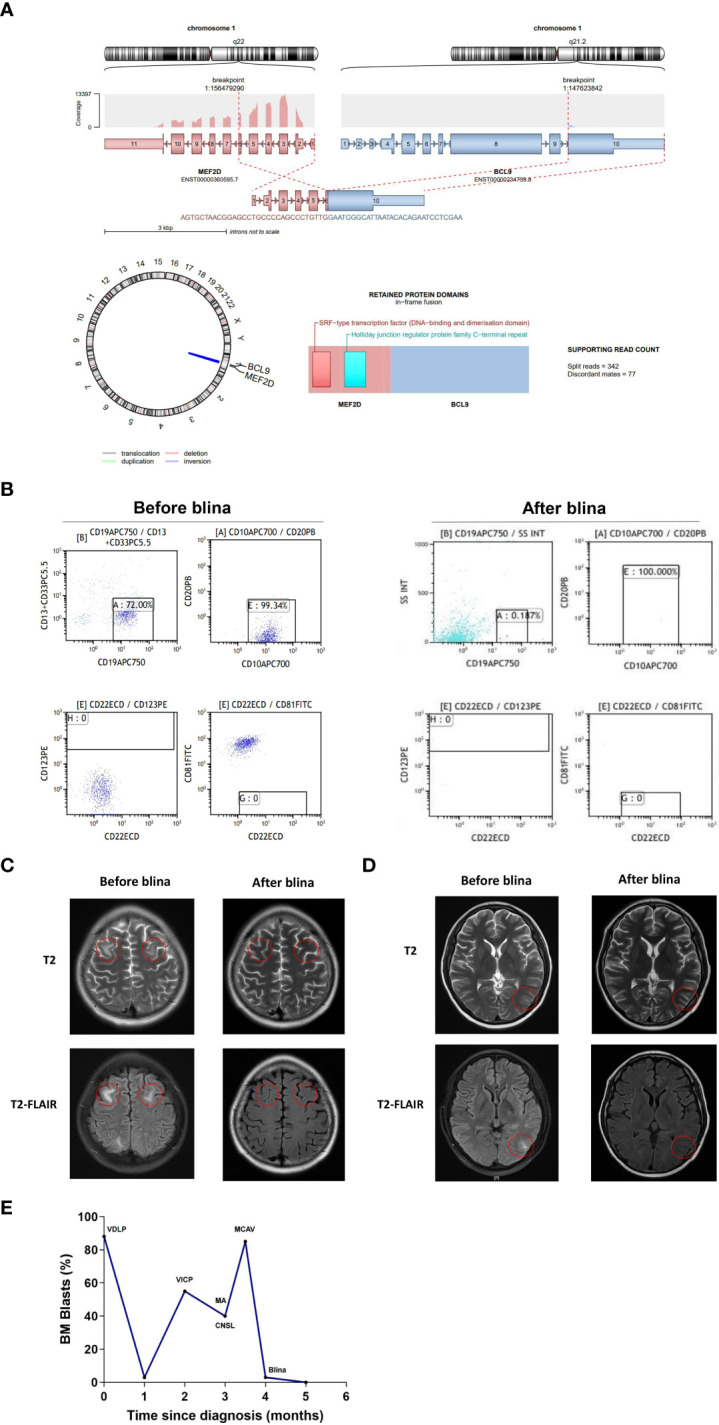
Clinical data of case 2. **(A)** Schematic diagram of the *MEF2D::BCL9* fusion before and after blina. **(B)** Flow cytometry analysis of CSF before and after blina. **(C, D)** Brain MRI analysis before and after blina. **(E)** Timeline of treatments and responses of case 2. VDLP, vincristine, daunorubicin, L-asparaginase and prednisone; VICP, cyclophosphamide (750 mg/m^2^), idarubicin 10 mg per week for 4 times, vincristine 1.5 mg/m^2^ per week for 4 times, prednisone 60 mg/m^2^, once daily, 4 weeks; MA, methotrexate 1 g/m^2^, intravenously, d1, Ara-c 3 g/m^2^, intravenously, q12h d2-3; MCAV, Mitoxantrone liposomes 40 mg combined with CAV regimen (cladribine 5 mg/m^2^, d2-6, cytarabine 20 mg q12h, d2-8 and dose-escalation of venetoclax d1-7); Blina, Blinatumomab (9 µg d1-7, 28 µg d8-28); CNSL, central nervous system leukemia.

A 16-year-old male was admitted to the hospital because of fever. PB counts showed white blood cells 21.9×10^9^/L, hemoglobin 151 g/L, and platelets 70×10^9^/L. BM smear revealed 88.76% of blasts, which were CD10+/CD13-/CD19+/CD20-/CD22±/CD33-/CD34+/CD38+ by FCM. Cytogenetics was 47, XY, add(1)(q42), +8 [3]/47, idem, add(5)(q13), add(9)(p22)[12]/46, XY[5]. *NRAS* p.Gly13Arg, p.Gln61Arg, p.Gln61Lys mutations were identified by NGS. *MEF2D::BCL9* fusion gene was detected by RNA sequencing (**Figure 2A**). He was diagnosed with B-ALL. Induction chemotherapy with vincristine, daunorubicin, L-asparaginase and prednisone was initiated, together with intrathecal chemotherapy with dexamethasone, cytarabine and methotrexate to prevent CNS leukemia. A brief CR was achieved, followed by early relapse with 55% of blasts. Unfortunately, the patient failed re-induction chemotherapy with cyclophosphamide (750 mg/m^2^) for one day, idarubicin 10 mg/m^2^ per week for 4 times, vincristine 1.5 mg/m^2^ per week for 4 times, prednisone 60 mg/m^2^, once daily, for 4 weeks. He complicated slurred speech and facial paralysis. Massive blasts were detected in the CSF, which were CD19 positive (**Figure 2B**). CNSL (CNS-3) was confirmed. Intermittent intrathecal chemotherapy was applied. MA regimen (methotrexate 1 g/m^2^, intravenously, d1, Ara-c 3 g/m^2^, intravenously, q12h d2-3) was administered. The patient complicated vomiting, headache and seizure. Meanwhile, slurred speech and facial paralysis were worsened. The patient got 1 point in facial movements and visual fields, respectively. The final NIHSS score was 2. BM morphology showed 85% of blasts, which were CD10+/CD19+/CD22+/CD34+/cCD79a+. Mitoxantrone liposomes (40 mg) combined with CAV regimen (cladribine 5 mg/m^2^, d2-6, cytarabine 20 mg q12h, d2-8 and dose-escalation of venetoclax d1-7) was further initiated. BM evaluation showed no blasts, with 3.48% of MRD by FCM. However, there was no improvement in speech or vision. The brain MRI showed bilateral frontal lobe (**Figure 2C**) and left occipital lobe (**Figure 2D**) swelling with abnormal signals, indicating the persistence of CNS leukemia. Blinatumomab (9 µg d1-7, 28 µg d8-28) was subsequently initiated. Symptoms of facial paralysis, blurred vision and headache relieved gradually. The NIHSS score decreased to 0. One week after completion of blinatumomab, BM analysis showed 1% of blasts with MRD of 1.6×10^-5^ and negativity for *NRAS* mutation and *MEF2D::BCL9* fusion gene. There were no blasts in the CSF detected by FCM. MRI of the brain showed disappearance of abnormal signals in both frontal lobe and decrease of abnormal signals in the left occipital lobe. Then, the patient received haploidentical allo-HSCT from his father and he has remained complete remission for 6 months after blinatumomab treatment. The treatment process of case 2 is depicted in **Figure 2E**.

## 4 Discussion

*MEF2D*-rearranged B-ALL is a new entity in the International Consensus Classification (ICC) of Myeloid Neoplasms and Acute Leukemia (12). And *MEF2D::BCL9* is the most common *MEF2D*-rearrangement. In the largest study on genetic analysis of recurrent *MEF2D* fusions in ALL, the incidence of *MEF2D::BCL9* was 2.86% (16/560) (13). Both *MEF2D* and *BCL9* are located at 1q21.2-22 and the *MEF2D::BCL9* fusion is frequently resulted from cryptic interstitial insertion on cytogenetic analysis. *NRAS* mutations were frequently found in patients with *MEF2D::BCL9* (37.5%, 6/16). The *MEF2D::BCL9* fusion is more potent in activating expression than wild-type *MEF2D* and confers hematopoietic self-renewal. *MEF2D::BCL9*-positive patients were characterized as being older in age of adolescents, being resistant to chemotherapy, having very early relapse (14), and may be a candidate for novel molecular targeting therapy. Patient 2 in this study was 16 years old, had three *NRAS* mutations and presented with resistance to chemotherapy and very early relapse, all of which are consistent with the clinical features described above. Fortunately, after blinatumomab treatment, the patient achieved complete hematologic and CNS remission. Our results show that patients with *MEF2D::BCL9* rearranged-B-ALL can benefit from blinatumomab.

In this study, blinatumomab was observed to successfully induce both complete hematologic and CNS remission in 2 patients with R/R B-ALL. Olverembatinib is a novel third-generation TKI approved in China for CML in 2021, which demonstrated low permeability through BBB in preclinical studies (15). Therefore, we believe that the remission of CNS leukemia in case 1 can be attributed to blinatumomab. Recently, it was found that T-cells can migrate through the meningeal lymphatics to the CNS (16), which may kill CNS leukemic cells through T-cell induced cytotoxicity. Our group treated 4 B-ALL patients with CNS leukemia using anti-CD19 CAR T-cell therapy, the results showed an overall response rate of 100% (17). In another retrospective multi-center study, anti CD19 CAR T-cell therapy used to treat CNS leukemia showing that 85.4% of 48 patients achieved CR (18). Blinatumomab has a molecular weight of approximately 54 kDa, which is significantly larger than the size of drugs allowed to directly pass through the BBB (<400 Da) (19). Theoretically, blinatumomab can directly penetrate into the CNS only when BBB is disrupted (20). With an *in vitro* migration model, we found that blinatumomab engaged T-cells exhibited the ability to migrate and kill the CD19 positive cells (**Supplementary Figures 1, 2**), so we hypothesized that T cells binding to blinatumomab migrated to the CNS, allowing blinatumomab to identify and kill the CD19 positive leukemic cells in the CNS of these two patients.

To our knowledge, this is the first report on the efficacy of blinatumomab in treating CNS leukemia, of the CSF and the cerebral parenchymal involvement. From clinical efficacy and reasonable experimental evidence, we believe that blinatumomab can migrate to CNS carried by T cells. Patient 1 is expected to attain long-term survival through maintenance therapy with blinatumomab and third-generation TKIs. The administration of blinatumomab provided the opportunity for allo-HSCT in patient 2. The duration of response to blinatumomab in both patients requires regular follow-up. Because we have reported few cases, additional large and prospective clinical trials are needed to demonstrate the efficacy of blinatumomab in anti-central nervous system leukemia, which may expand the indications for blinatumomab.

## Data availability statement

The raw data supporting the conclusions of this article will be made available by the authors, without undue reservation.

## Ethics statement

Written informed consent was obtained from the individual(s), and minor(s)’ legal guardian/next of kin, for the publication of any potentially identifiable images or data included in this article.

## Author contributions

NX, S-BL, T-TZ performed experiment and analyzed data. H-YC, HC, W-JG, C-LW, S-MH collected the clinical data. H-YC wrote the manuscript, which was approved by all the authors. H-PD, S-LX, C-SQ helped perform the analysis with constructive discussions. All authors contributed to the article and approved the submitted version.
