# Supplementary material for: Case Report: Blinatumomab therapy for the treatment of B-cell acute lymphoblastic leukemia patients with central nervous system infiltration
Source: Front Immunol. 2023 Apr 18;14:1181620. doi: 10.3389/fimmu.2023.1181620 (PMC10151792; doi:10.3389/fimmu.2023.1181620)
Supplement: Supplementary file 1 [file DataSheet_1.docx]

Supplementary Material

Case report: Blinatumomab therapy for the treatment of B-cell acute lymphoblastic leukemia patients with central nervous system infiltration

Han-Yu Cao1,2†, Hui Chen3†, Song-Bai Liu4†, Wen-Jie Gong1,2†, Chong-Sheng Qian1,2, Tong-Tong Zhang1,2, Chao-Ling Wan1,2, Si-Man Huang1,2, Nan-Xu5, Hai-Ping Dai1,2*, Sheng-Li Xue1,2*

*** Correspondence:** Professor Sheng-Li Xue: slxue@suda.edu.cn

Dr. Hai-ping Dai: daihaiping8@126.com

# Supplementary Methods

Transwell migration assay

T cells were sorted from peripheral blood samples of 3 B-ALL patients, using CD3 antibody-coated magnetic beads (Miltenyi, Germany). Then the T-cells (range from 2.18×10^5^ to 2.62×10^5^ cells) were seeded into a transwell insert with a polyethylene terephthalate membrane pore size of 8 μm (Thermo Fisher, MA, USA) in a 24-well plate, respectively. Raji cells (ATCC, USA) were seeded in the lower chamber (5×10^5^ cells/well). Each sample was divided into 2 groups as shown in Supplementary Figure 1A. Blina (1µg/ml) were incubated with T cells for an hour at 4°C before seeding into the upper chamber. The 24-well plates were incubated at 37°C, 5% CO^2^ for 18 hours. Then the upper chambers were taken out. Cell numbers in the upper and lower chambers were counted, respectively.

Flow cytometry analysis

T-cells were separated, grouped and treated as indicated in Supplementary Figure 1A. Cells in the lower chamber were collected and prepared for flow cytometry analysis. PE-Cy7-A CD20, PerCP-Cy5.5-A CD4 and or CompAPC-A CD8 antibodies (Proteintech, USA) were used. Flow cytometry analysis was performed on the cells according to the protocols of our institute.

# Supplementary Results

Results of transwell migration assay

The results showed that T-cells and T-cells with blinatumomab seeded in the upper chamber penetrated into the lower chamber (Supplementary Figure 1).

Results of flow cytometry

CD20 positive CD4+ or CD8+ T cells were detected in the lower chamber, which indicated that blinatumomab engaged T cells migrated to the lower chamber (Supplementary Figure 2).

# Supplementary Figures and Tables

## Supplementary Figures

##
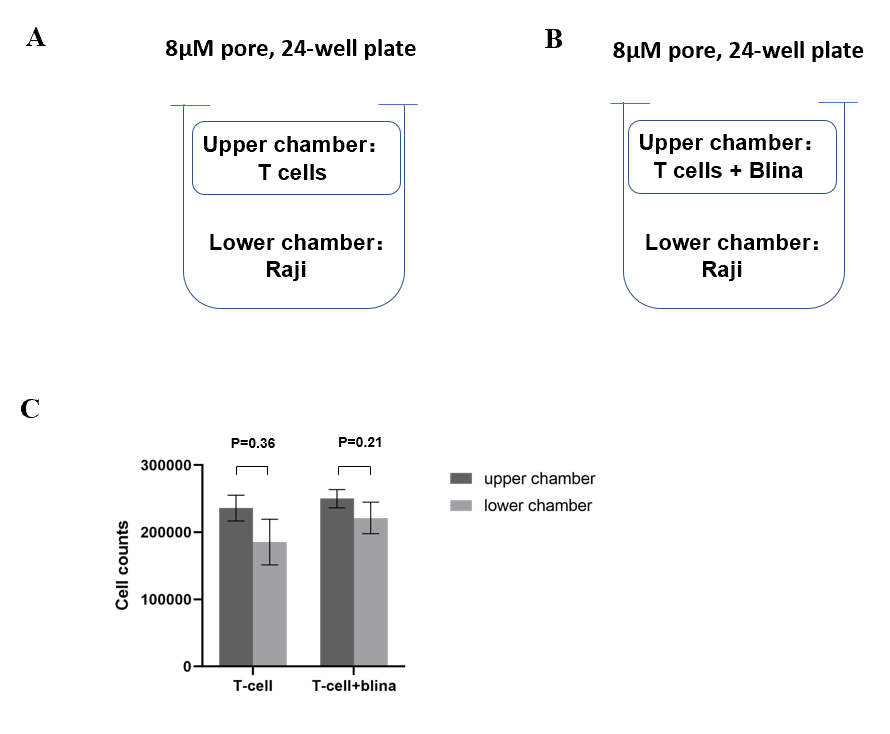


## Supplementary Figure 1. Transwell migration assay. (A, B) The schematic diagram of transwell migration assay. (C) Cell counts of the upper and lower chambers of each group in the transwell migration assay.


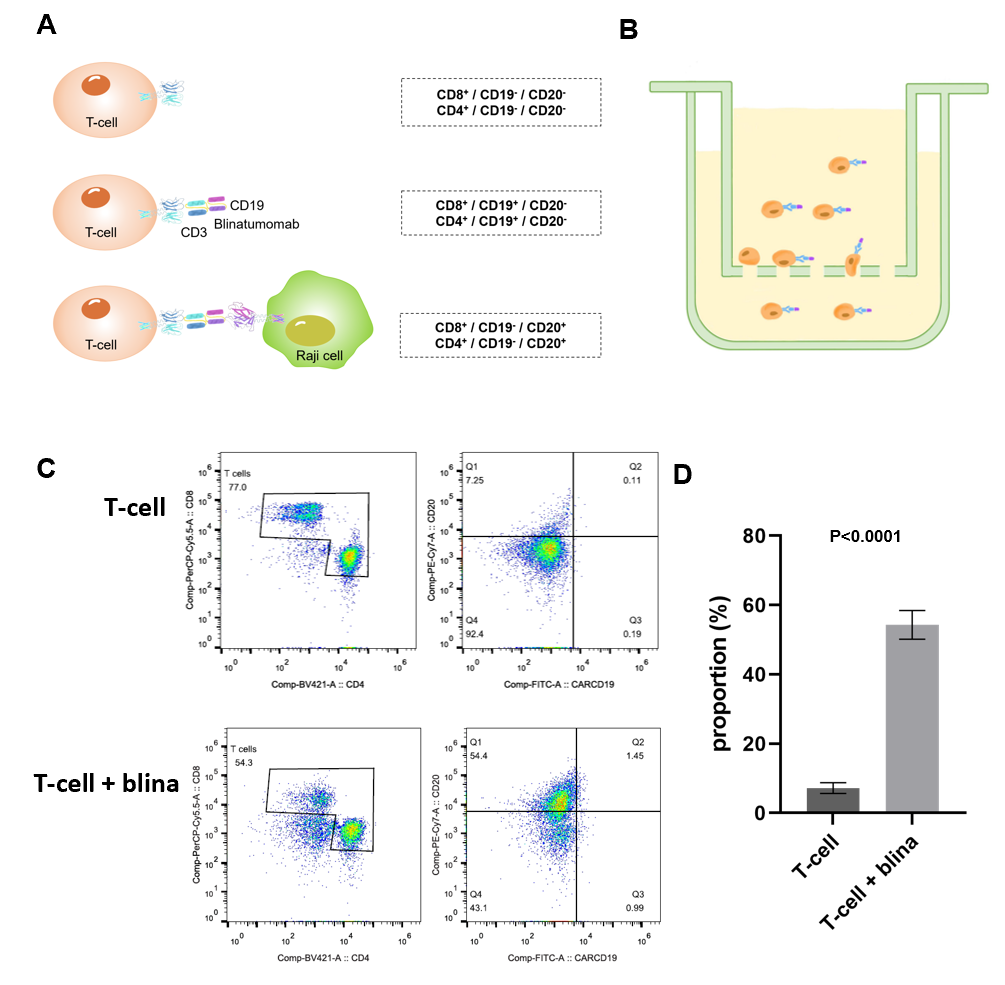
**Supplementary Figure 2.** Flow cytometry assays. (A) Possible binding forms of T cells to blina or Raji cells in migration assays. (B) The schematic diagram of blina engaged T-cells across to the lower chamber in the transwell migration assay. (C) Raji cells combined with Blinatumomab engaged T cells were detected in the lower chamber by flowcytometry. (D) The proportion of CD4+/CD19+/CD20+ cells together with CD8+/CD19+/CD20+ cells in the lower chamber detected by flow cytometry.
